# First-phase ejection fraction is associated with myocardial fibrosis in the pressure overloaded heart

**DOI:** 10.3389/fcvm.2025.1603082

**Published:** 2025-07-23

**Authors:** Shukun He, Jingrong Jiang, Yanting Zhang, Tianshu Liu, Wenhui Deng, Yuji Xie, Wenqu Li, Yuting Tan, Lingyun Fang, Jing Zhang, Lin He, Qiaofeng Jin, Yuman Li, Li Zhang, Phil Chowienczyk, Mingxing Xie, Haotian Gu, Jing Wang

**Affiliations:** ^1^ Department of Ultrasound Medicine, Union Hospital, Tongji Medical College, Huazhong University of Science and Technology, Wuhan, China; ^2^ Hubei Province Key Laboratory of Molecular Imaging, Union Hospital, Tongji Medical College, Huazhong University of Science and Technology, Wuhan, China; ^3^ Clinical Pharmacology, St Thomas’ Hospital, King’s College London, London, United Kingdom

**Keywords:** first-phase ejection fraction, myocardial fibrosis, ejection fraction, myocardial strain, pressure overload

## Abstract

**Background:**

First-phase ejection fraction (EF1) has been recently demonstrated to sensitively detect early cardiac systolic dysfunction. However, the value of EF1 in predicting myocardial fibrosis (MF) has not been investigated. This study aimed to explore the relationship between EF1 and MF in the pressure overloaded heart.

**Methods:**

The pressure overloaded heart was induced by minimally invasive transverse aortic constriction (MTAC) in rats. Rats in the sham and MTAC groups were equally divided into different time points for examination, respectively. Echocardiography was conducted to validate the success of MTAC model and measure cardiac systolic function parameters. Subsequently, rat hearts underwent Masson's staining to measure the degree of MF.

**Results:**

Compared with sham group rats, MTAC group rats exhibited a significantly progressive impairment in EF1 starting from the 2nd week over observational period (*P* < 0.01), while GLS, GCS, GRS and EF showed no significant difference until the 3rd week and 4th week respectively. MF strongly correlated with EF1 (*r* = −0.78, *P* < 0.001), modestly with GLS, GCS and GRS (*r* = −0.65 to −0.51, *P* < 0.001), and weakly with EF (*r* = −0.42, *P* < 0.05). Receiver operating characteristic curve indicated that EF1 exhibits excellent performance in the detection of moderate and severe MF (area under the curve = 0.87, *P* < 0.001).

**Conclusions:**

EF1 represents a highly sensitive and non-invasive marker for the early detection of cardiac systolic dysfunction and emerges as a promising indicator for the identification of MF in the early stage of pressure overloaded heart.

## 1 Introduction

Myocardial fibrosis (MF) is a common pathophysiologic response to stress, injury or aging, which is characterized by excessive deposition of extracellular matrix (ECM) in the cardiac interstitium (1, 2). MF can increase myocardial stiffness and diminish myocardial compliance, precipitating cardiac dysfunction and eventual heart failure (HF) (2–4). Previous studies have shown that the MF degree is highly correlated with the long-term mortality in patients with cardiac dysfunction (5, 6), even with proper treatment, MF also affects the clinical course and prognosis of patients with cardiac dysfunction (7–9). Consequently, early detection of MF and timely intervention are particularly important for patients with cardiac dysfunction (7, 9, 10).

Subendocardial myocardial biopsy is the gold standard for diagnosing MF, yet its clinical utility is hindered by invasiveness and associated complications (11, 12). In recent years, cardiac imaging has gained prominence because of its non-invasive and repeatable MF diagnosis capabilities. Echocardiography plays an indispensable role in MF and cardiac function assessment, owing to its non-invasiveness, high reproducibility, and cost-effectiveness. However, traditional echocardiographic parameters such as the left ventricular ejection fraction (EF) lack sensitivity in capturing MF progression and dynamic changes in cardiac systolic function, particularly in left ventricular pressure overload states (13). Left ventricular global longitudinal strain (GLS) has shown associations with MF and adverse events in patients with severe AS (14–16), while its diagnostic potential for early MF in patients with cardiac dysfunction warrants further investigation.

Notably, our team found that the new echocardiographic parameter, first phase ejection fraction (EF1), represented the EF up to the time of peak aortic flow velocity, which could sensitively detect the early systolic dysfunction in AS and hypertension patients with normal EF and GLS (17, 18). And we have proved that EF1 is correlated with cardiac magnetic resonance markers of left ventricular MF, which indicates that EF1 may have certain diagnostic value for left ventricular remodeling in AS patients (19, 20). Nevertheless, it remains unclear whether EF1 is capable of detecting the onset of left ventricular systolic dysfunction during the progression of cardiac impairment. Furthermore, the potential of EF1 as a sensitive marker for the early stages of MF in the course of cardiac dysfunction development has yet to be fully validated.

The aim of this study was: (1) to determine the patterns of EF1 change in the progression of pressure overload induced cardiac dysfunction, (2) to explore the relationship between EF1 and MF in pressure overload induced cardiac dysfunction rats.

## 2 Methods

### 2.1 Ethics

The animal study was approved by the Institutional Animal Care and Use Committee at Tongji Medical College, Huazhong University of Science and Technology (IACUC Number: 3301), and this study was reported in accordance with the ARRIVE guidelines.

### 2.2 Animals and MTAC surgery

Male Sprague-Dawley rats aged six weeks (weight 200–220 g) were obtained from Hubei Biont Biological Technology Co., Ltd. The rats were divided into two groups: MTAC group (*n* = 35), sham group (*n* = 30). Before the surgical procedures, the rats were anaesthetized with 1.5%–2% isoflurane. The MTAC group rats underwent minimally invasive transverse aortic constriction (MTAC) by placing a ligature around the transverse aorta using a 22G needle and a 4–0 silk thread (21, 22). Sham group rats underwent the same procedure as the MTAC group, except without ligature. Buprenorphine (0.05 mg/kg) was administrated post-operatively to the sham and MTAC group rats.

Rats were randomly aliquoted into different observation time groups (MTAC group, *n* = 7; sham group, *n* = 6) for echocardiography assessment. Subsequently, the selected rats were euthanized by isoflurane overdose followed by histological evaluation. It should be noted that, the measurements represent independent groups across timepoints rather than repeated measures from the same animals.

### 2.3 Echocardiography

**Figure 1:**
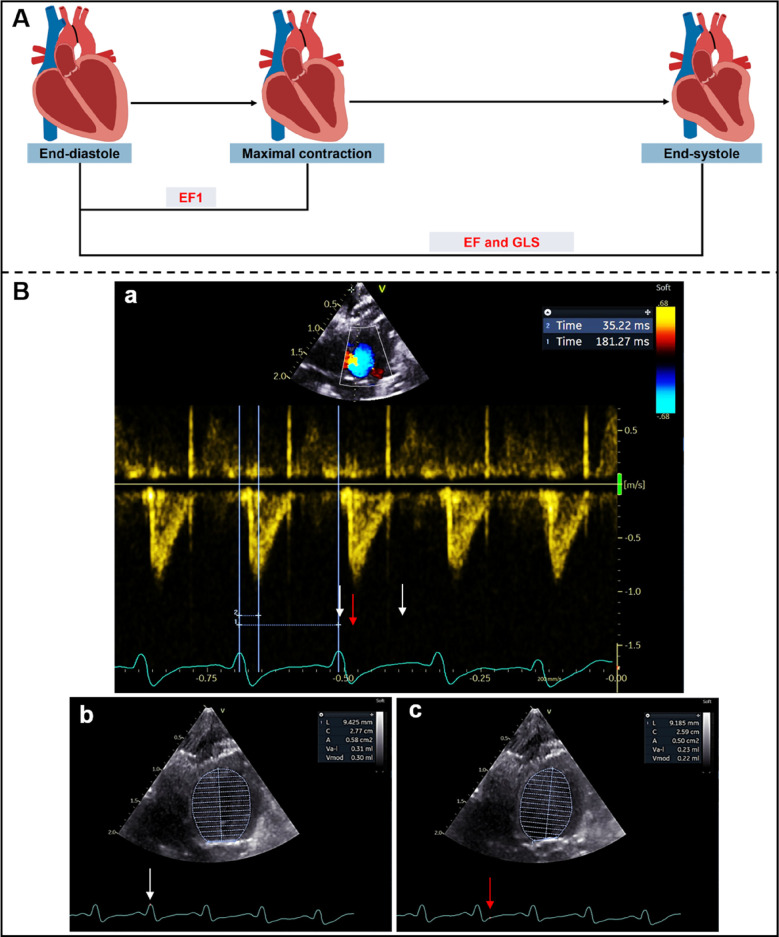
**(A)** Schematic diagram of left ventricular myocardial movement during systole; **(B)** EF1 was measured using Simpson's method from apical four-chamber view. ECG R-R interval (181 ms) (white arrows) and R to time of peak aortic flow velocity (35 ms) (red arrow) were measured from aortic valve continuous-wave Doppler **(a)**, end-diastolic volume (0.30 ml) **(b)** and volume at time of aortic valve flow velocity (0.22 ml) **(c)** were used to calculate EF1 (27%).

Transthoracic echocardiography was performed using a Vivid E95 ultrasound system with a 12S probe and an EchoPAC workstation (Version 204, GE Medical Systems, Milwaukee, WI, USA). Briefly, rats were anesthetized with 1.5%–2% isoflurane before echocardiography and positioned in the supine position at room temperature. Continuous-wave Doppler imaging was performed to determine the peak flow velocity and mean pressure gradient (MPG) after operation at the aortic arch, and to measure the time to peak aortic valve flow (TAVPF) and ECG R-R interval in the apical five-chamber view. Cardiac systolic function was measured in rats at different time points. The end-diastolic volume (EDV), left ventricular volume at the time of peak AV flow (V1) and end-systolic volume (ESV) were measured by Simpson's method at the apical four-chamber view. EF1 was calculated as the percentage change in left ventricular volume from end-diastole to the time of peak aortic flow velocity [EF1 = (EDV-V1)/EDV × 100%] (Figures 1A,B). EF was calculated as EF = (EDV − ESV)/EDV × 100%.

Left ventricular global myocardial strain was measured using 2D speckle tracking echocardiography (TomTec 3.1) with a frame rate of ≥164 frames per second. The endocardial border was initially defined at the end of diastole and systole. Automated tracking was subsequently performed over the entire cardiac cycle. GLS, global circumferential strain (GCS) and global radial strain (GRS) were averaged from three cardiac cycles from the apical four-chamber and left ventricular short-axis papillary muscle horizontal views. GLS and GCS values were negative; however, we used absolute GLS and GCS values for intuitive interpretation, with higher absolute values (more negative) representing better cardiac function.

### 2.4 Histological examination

After euthanasia, body weight (BW), heart weight (HW) and tibial length (TL) were quickly measured to calculate the heart weight-to-body weight (HW/BW) and heart weight-to-tibial length (HW/TL) to assess the extent of left ventricular hypertrophy (21, 23). The hearts were transversely bisected between the atrioventricular sulcus and the cardiac apex. Samples were fixed in 4% formaldehyde, embedded in paraffin, and sectioned into 5-μm-thick transverse slices (24). Haematoxylin and eosin (H&E) staining was performed to assess the morphology of cardiomyocytes, and the stained sections were scanned using a high-capacity digital slide scanner (3DHISTECH, Hungary). The cross-sectional area (CSA) of cardiomyocytes was measured using CaseViewer software. Masson's trichrome staining was performed to assess the degree of MF, and the stained sections were scanned using a high-capacity digital slide scanner at 0.8× and 8× magnifications (3DHISTECH, Hungary). For each heart, a single transverse slice was histologically evaluated to quantify degree of myocardial fibrosis (24, 25). Three randomly selected fields per histopathological section were analyzed. The section-level fibrosis ratio was calculated as the mean value derived from these three fields (26). Collagen volume fraction (CVF) was calculated as the ratio of collagen-positive (blue) area to total myocardial tissue area, providing an objective and reproducible measure of fibrosis burden. CVF measurements were performed in Image J software (24–27).

### 2.5 Observer variability

Ten rats were randomly selected to assess the inter-observer and intra-observer variability of the MF and EF1. Intra-observer variability was evaluated by a single investigator (S He) at intervals of four weeks between measurements. Inter-observer reproducibility was assessed by a blinded second observer (J Jiang). Observer variability analyses were conducted using the interclass correlation coefficient (ICC) and Bland-Altman analysis.

### 2.6 Statistical analysis

Continuous variables were expressed as mean ± standard deviation (mean ± SD), while categorical variables were expressed as percentage (%). Two-way analysis of variance (ANOVA) with Sidak's multiple comparisons test was used to test the difference between sham and MTAC groups at the same time point. Pearson correlation coefficients were used to analyze the correlations between echocardiographic functional parameters and MF. MTAC group rats (*n* = 35) were categorized into three groups according to tertiles of MF (mild, moderate and severe) (28, 29). Receiver operating characteristic (ROC) curves were conducted to determine the diagnostic efficiency of various parameters in detecting moderate and severe MF (*n* = 24). Comparisons of correlation coefficients and areas under the ROC curves (AUCs) were performed with the use of MedCalc version 18.2.1 (MedCalc Software, Ostend, Belgium) (28). All data were analyzed using Graphpad Prism version 8.0 (GraphPad, San Diego, CA, USA), SPSS 26.0 (IBM Corporation, Armonk, NY, USA) and MedCalc version 18.2.1 (MedCalc Software, Ostend, Belgium). Statistical significance was set at *P* < 0.05.

## 3 Results

### 3.1 MTAC model validation and cardiac remodeling

**Figure 2:**
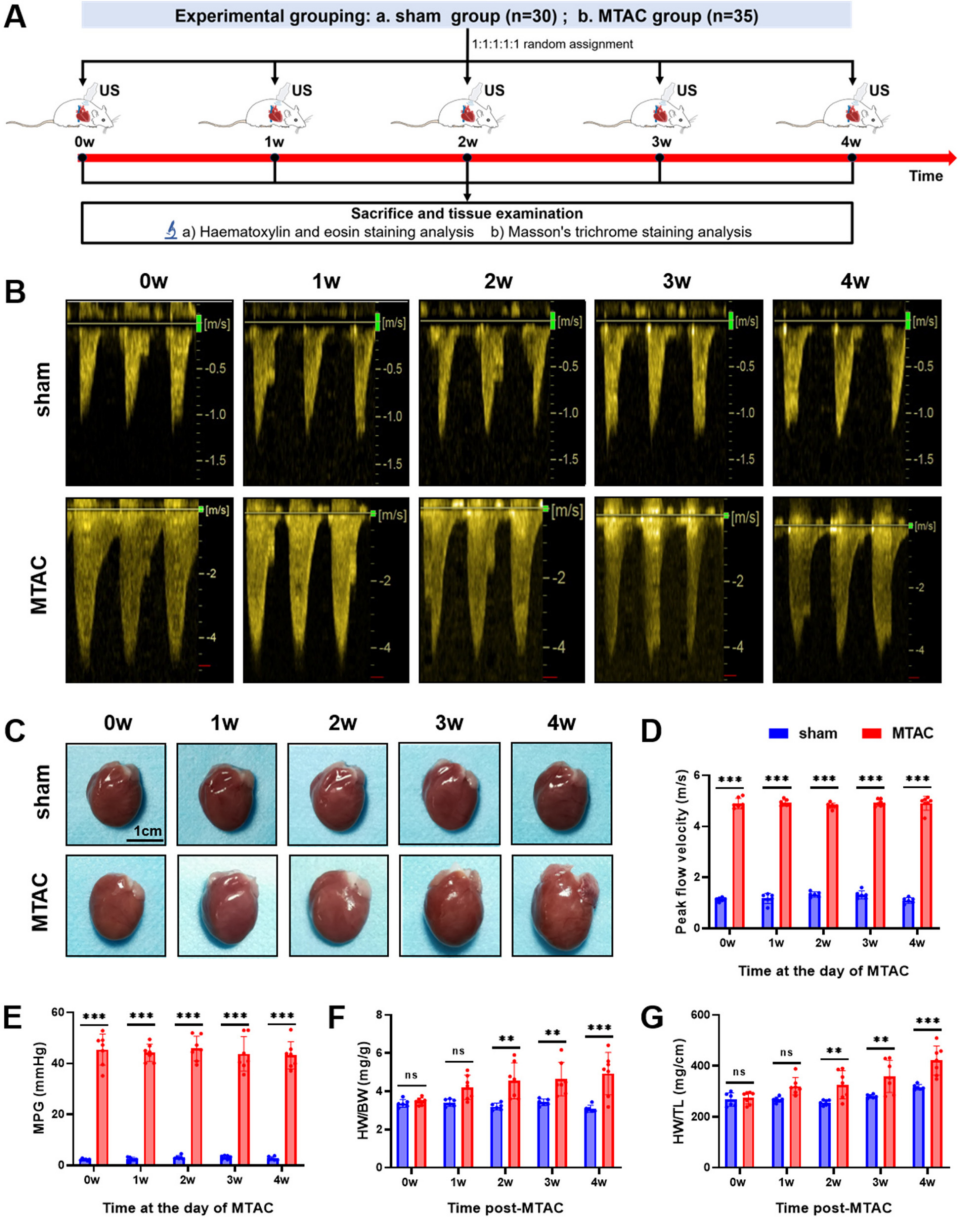
**(A)** Experimental grouping and time-line of echocardiography and histological examination of rats; **(B)** continuous-wave spectral Doppler of aortic arch at the day of MTAC operation; **(C)** representative heart images of rats, scale bar = 1 cm; **(D)** aortic arch peak flow velocity; **(E)** MPG, mean pressure gradient; **(F)** HW/BW, heart weight to body weight ratio; **(G)** HW/TL, heart weight to tibia length ratio. (**, *P* < 0.01; ***, *P* < 0.001; two-way ANOVA with Sidak's multiple comparisons test was used for statistical analysis). *n* = 6 in sham group and *n* = 7 in MTAC group.

Echocardiography was performed to assess the degree of aortic arch narrowing after MTAC operation and to evaluate cardiac function at 0–4 weeks postoperatively, then rat hearts were removed for pathological testing (Figure 2A). Continuous - wave Doppler showed that the peak flow velocity and MPG of aortic arch in the MTAC group rats significantly increased after the operation compared to the sham groups rats (*P* < 0.001) (Figures 2B–E). Compared with the sham group rats, the MTAC group rats exhibited progressively increased heart size, HW/BW and HW/TL from the 1st week after the operation, and there was a significant increase of HW/BW and HW/TL at the 2nd week after the operation (4.6 ± 0.94 mg/g vs. 3.21 ± 0.18 mg/g, 326 ± 55 mg/cm vs. 257 ± 10 mg/cm, respectively, all *P* < 0.01) (Figures 2C–G).

### 3.2 Cardiac dysfunction

**Figure 3:**
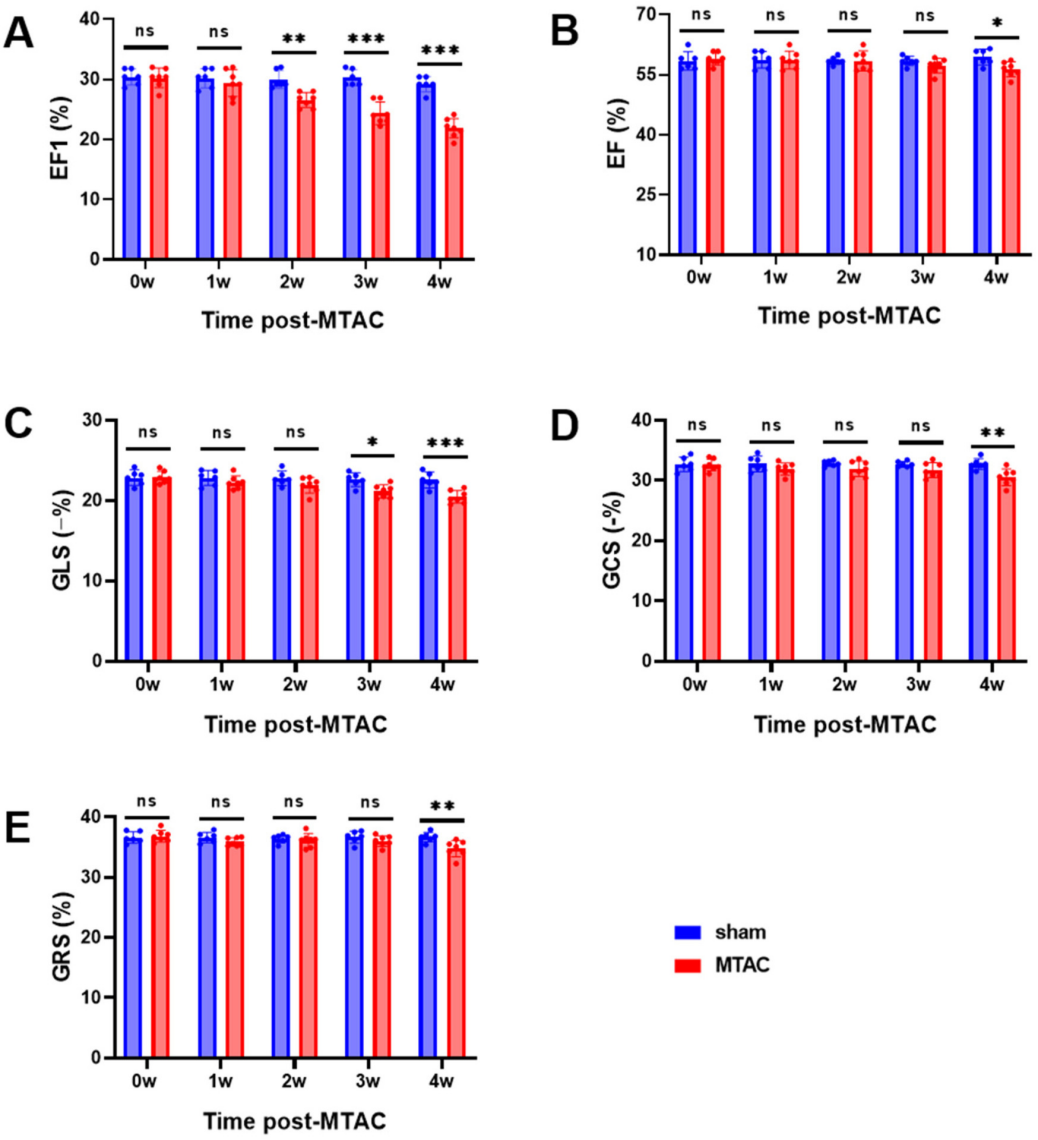
Echocardiographic parameters of cardiac systolic function. **(A)** EF1, first-phase ejection fraction; **(B)** EF, left ventricular ejection fraction; **(C)** GLS, global longitudinal strain; **(D)** GCS, global circumferential strain; **(E)** GRS, global radial strain. Graphs represent mean ± SD. (*, *P* < 0.05; **, *P* < 0.01; ***, *P* < 0.001; two-way ANOVA with Sidak's multiple comparisons test was used for statistical analysis). *n* = 6 in sham group and *n* = 7 in MTAC group.

Compared with the sham group rats, MTAC group rats showed significant reduction of EF1 at the 2nd week after the operation (26.6 ± 1.3% VS. 30.0 ± 1.5, *P* < 0.01) (Figure 3A), while EF, GLS, GCS and GRS showed no significant difference between the two groups at the 2nd week (all *P* > 0.05 for all). With the extension of observation time, GLS in the MTAC group rats did not show a significant reduction until the 3rd week (mean decrease 0.86% between the two groups at the 2nd week, *p* = 0.36; mean decrease 1.5% between the two groups at the 3rd week, *p* = 0.02), EF, GCS and GRS in the MTAC group rats did not show a significant reduction until the 4th week (Figures 3B–E).

### 3.3 Cardiac hypertrophy and fibrosis

**Figure 4:**
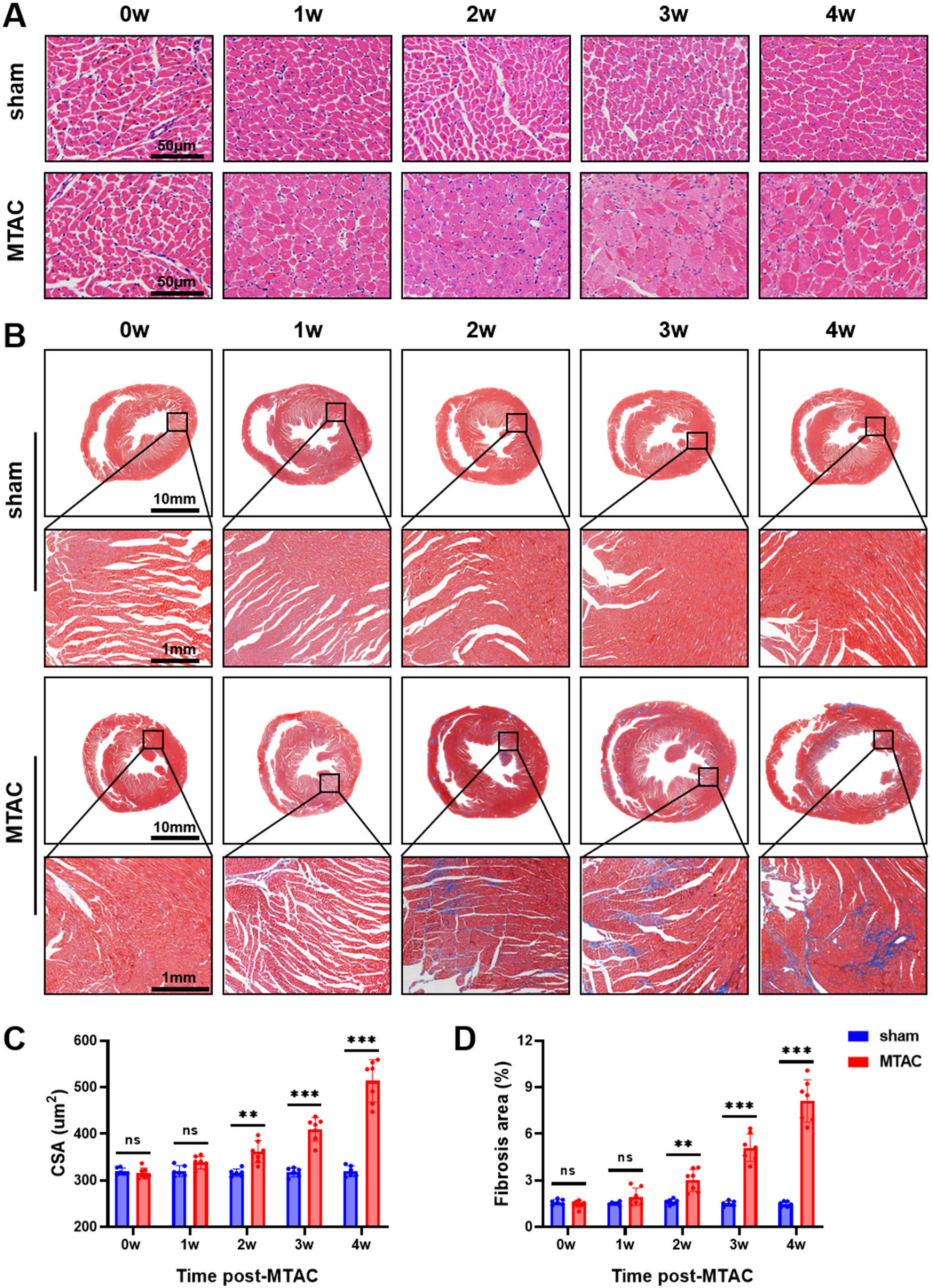
**(A)** Pathology staining of myocardial tissue. Haematoxylin and eosin (H&E) staining of myocardial tissue, scale bar = 50 μm; **(B)** Masson's trichrome staining of myocardial tissue; **(C)** the cross-sectional area (CSA) of myocardial cells; **(D)** Deposition of myocardial interstitial collagen, scale bar = 10 mm and 1 mm respectively. Graphs represent mean ± SD. (**, *P* < 0.01; ***, *P* < 0.001; two-way ANOVA with Sidak's multiple comparisons test was used for statistical analysis). *n* = 6 in sham group and *n* = 7 in MTAC group.

Compared with the sham group rats, MTAC group rats showed significant increase of CSA of cardiomyocytes at the 2nd week after the operation (362 ± 24 μm^2^ VS. 316.4 ± 8.3 μm^2^, *P* < 0.01), and this increase continued to show an upward trend (Figures 4A,C). Additionally, the content of myocardial interstitial collagen fibers in MTAC group rats also increased at the 2nd week after the operation (3.0 ± 0.72% VS. 1.62 ± 0.18%, *P* < 0.01), and continued to increase over time (Figures 4B,D).

### 3.4 Correlation of MF and CSA with cardiac function measurements

**Figure 5:**
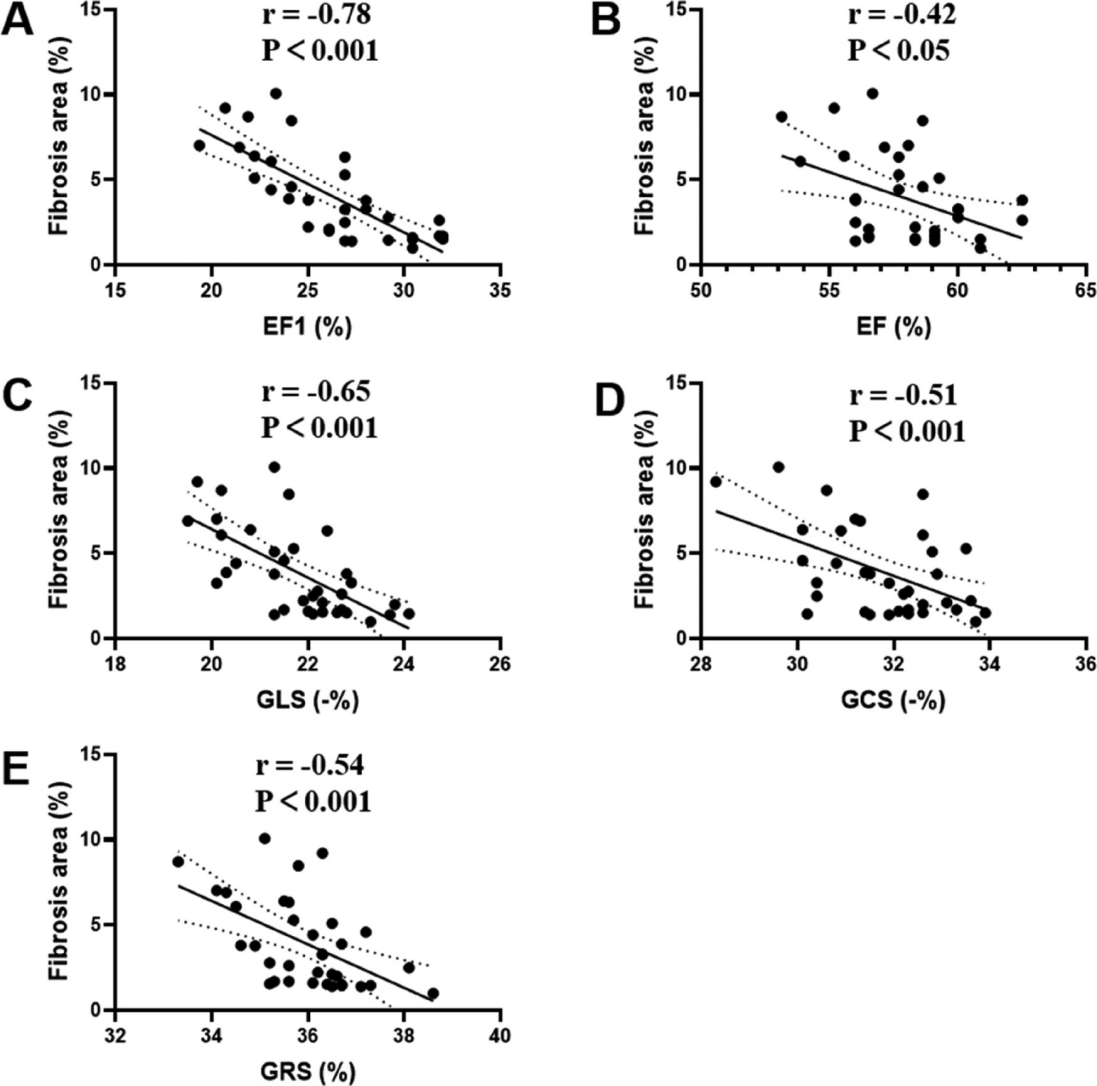
Correlation analyses between MF and EF1 **(A)**, EF **(B)**, GLS **(C)**, GCS **(D)**, GRS **(E)** in MTAC group rats. EF1, first-phase ejection fraction; EF, left ventricular ejection fraction; GLS, global longitudinal strain; GCS, global circumferential strain; GRS, global radial strain. *n* = 35.

Among the echocardiographic cardiac function measures in the MTAC group rats, MF strongly correlated with EF1 (*r* = −0.78, *P* < 0.001), modestly with GLS (*r* = −0.65, *P* < 0.001), GCS (*r* = −0.51, *P* < 0.001) and GRS (*r* = −0.54, *P* < 0.001), and weakly with EF (*r* = −0.42, *P* < 0.05) (Figure 5). Significantly, the correlation of MF with EF1 was better than that of EF (*r* = −0.78 vs. *r* = −0.42, *P* < 0.05), but the differences were small and not statistically significant compared to the correlation of the remaining parameters with MF (*P* = 0.29 for GLS; *P* = 0.06 for GCS; *P* = 0.08 for GRS, respectively).

CSA strongly correlated with EF1 (*r* = −0.77, *P* < 0.001), modestly with GLS (*r* = −0.64, *P* < 0.001) and GRS (*r* = −0.53, *P* < 0.01), and weakly with GCS (*r* = −0.48, *P* < 0.01) and EF (*r* = −0.39, *P* < 0.05) (Supplementary Figure S1). Furthermore, the absolute value of standardized beta coefficient of MF and CSA was larger than that of EF1, which means that MF is more correlated with CSA than EF1 (Supplementary Table S1). In addition, CSA strongly correlated with HW/TL (*r* = 0.70, *P* < 0.001) and weakly with HW/BW (*r* = 0.40, *P* < 0.05) (Supplementary Figure S2).

### 3.5 Echocardiographic parameters for detecting the moderate and severe MF

**Figure 6:**
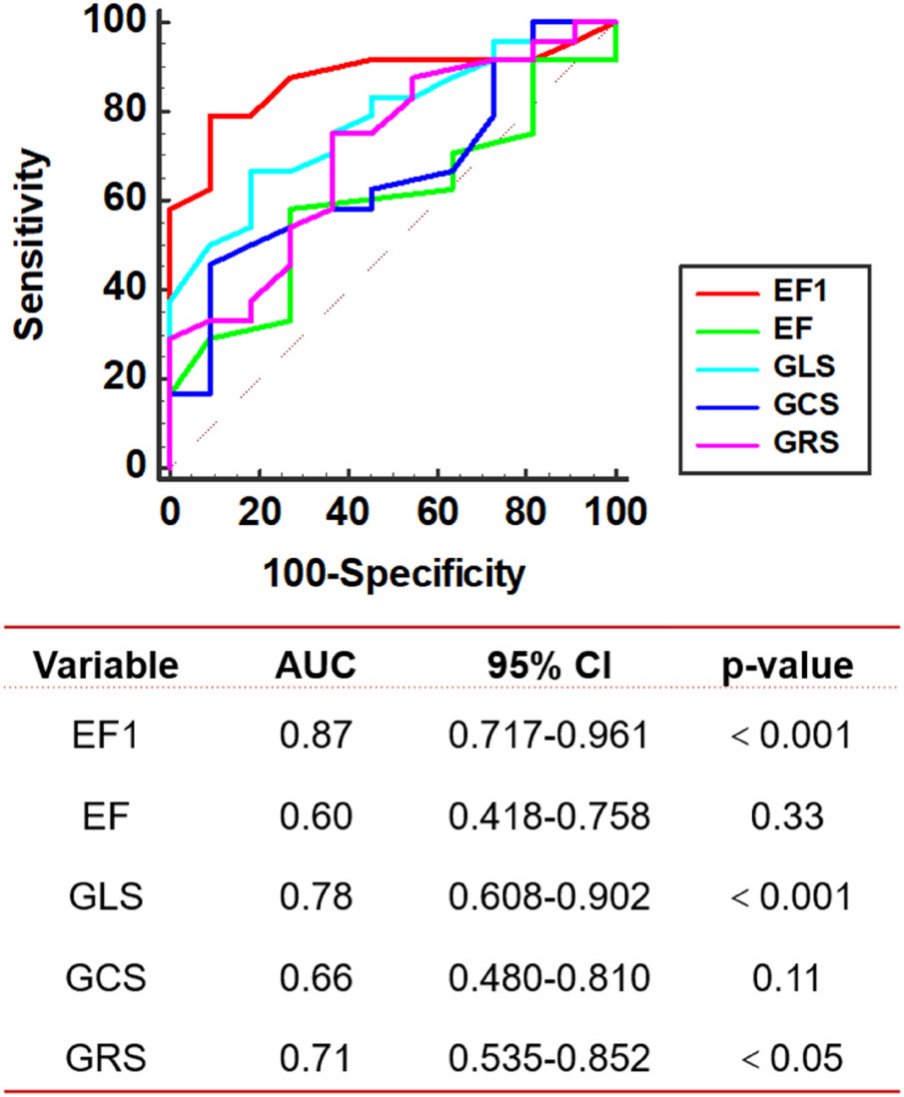
ROC curve analysis. ROC of echocardiographic parameters for detecting the moderate and severe MF in MTAC group rats. AUC, area under the curve; EF1, first-phase ejection fraction; EF, left ventricular ejection fraction; GLS, global longitudinal strain; GCS, global circumferential strain; GRS, global radial strain. *n* = 35.

The 35 rats in MTAC group were divided into 2 groups according to the tertiles of histologic MF (mild, moderate and severe). The mean value for the degree of MF was 1.49 ± 0.19% in the mild group and 5.0 ± 2.2% in the moderate and severe group. EF1, EF, GLS, GCS and GRS were entered into ROC analysis to evaluate the moderate and severe MF. The ROC analysis showed that the best cutoff value of EF1 for detecting the moderate and severe MF was 27% (AUC = 0.87, *p* < 0.001; sensitivity, 79%; specificity, 91%), and the optimal cutoff value of GLS and GRS for detecting the moderate and severe MF was −22% (AUC = 0.78, *p* < 0.001; sensitivity, 67%; specificity, 82%) and 36% (AUC = 0.71, *p* < 0.05; sensitivity, 75%; specificity, 64%), respectively; EF and GCS did not show diagnostic value in identifying the moderate and severe MF (Figure 6). We found that the AUC of EF1 (0.87) was greater than that of EF (0.60, *p* < 0.01). And the AUC of EF1 exceeded those of GLS (0.78), GCS (0.66), and GRS (0.71), although the difference was not statistically significant (*P* > 0.05 for all).

### 3.6 Observer variability

The intra-observer and inter-observer reproducibility of MF and EF1 were presented in Supplementary Table S1. The ICCs of intra-observer and inter-observer for MF were 0.95 and 0.94 respectively (all *P* < 0.001), and for EF1 were 0.80 (*P* = 0.001) and 0.75 respectively (*p* < 0.001). Bland-Altman analysis demonstrated that MF and EF1 had small bias and narrow limits of agreement.

## 4 Discussion

To the best of our knowledge, this is the first study to demonstrate an association between EF1 and MF in a rat model of pressure overload induced cardiac dysfunction. This study also presents, for the first time, a progressive impairment of EF1 during the course of pressure overload induced cardiac dysfunction, along with a gradual increase in the degree of MF and cardiac remodeling.

### 4.1 Reduction of EF1 at early stage of cardiac dysfunction

A major finding of this study is the progressive impairment of early cardiac systolic function, as measured by EF1, in pressure overload-induced cardiac dysfunction during observational period. Our results demonstrated a significant reduction in EF1 at the 2nd week post-operation in the MTAC group rats and GLS decreased at the 3rd week, while EF, GCS and GRS did not show a significant decrease until the 4th week. This highlights EF1 as the most sensitive marker of systolic function compared to conventional parameters such as global myocardial strain and overall EF. EF1 represents the EF at the time to aortic peak aortic jet velocity, corresponding to active and early systole. As such, EF1 is a novel and sensitive index of early contractile performance, with relatively reduced dependence on loading conditions compared to conventional EF or GLS (17, 18). EF1 is particularly reflective of peak myocardial contractility, which may decline before global systolic measures become abnormal (18).

GLS, on the other hand, is calculated at end-systole, capturing the cumulative deformation of the myocardium over the entire systolic period. It is more strongly influenced by late systolic load and may thus be less sensitive to early impairments in contraction dynamics (18, 30). Under pressure overload conditions, the heart may preserve end-systolic deformation through compensatory mechanisms—such as prolonged contraction duration—even in the presence of subtle early dysfunction (31). This could account for a transient dissociation between EF1 and GLS, where EF1 declines first. Furthermore, although GLS is known to reflect subendocardial function, which is indeed vulnerable to early ischemia or fibrosis, the timing of deformation may still appear preserved due to compensation from mid-myocardial fibers or altered contraction timing. This nuance has been observed in clinical studies, where EF1 was found to be more sensitive than GLS for detecting early systolic dysfunction in patients with aortic stenosis or hypertension (20, 30, 32).

In addition, we found that GLS was decreased earlier than EF, GCS and GRS during the progression of pressure overload-induced cardiac dysfunction. Pressure overload results in cardiac remodeling and dysfunction although EF is preserved. The predominant involvement of the subendocardial fibers results in a GLS reduction, but EF, GCS and GRS are maintained because of the compensation of the unaffected midmyocardial and subepicardial fibers (33, 34).

Consequently, EF1 emerges as a more sensitive parameter for detecting early cardiac systolic dysfunction than EF or GLS. Our findings align with those of previous studies in patients with AS, where EF1 was progressively impaired with the progression of severity of AS, while over EF was preserved (20). The continuous decline in EF1 observed alongside the progression of cardiac dysfunction suggests that EF1 could serve as a valuable marker for the dynamic monitoring of cardiac systolic function in clinical settings.

### 4.2 Cardiac fibrosis and remodeling at early stage of cardiac dysfunction

In the present study, histological examination revealed a progressive increase in the CSA of cardiomyocytes and the formation of interstitial fibrosis as cardiac dysfunction advanced in the MTAC group rats. Remarkably, evident MF and adverse cardiac remodeling were observed as early as the 2nd week post-operation, indicating rapid pathological responses in response to pressure overload. Prolonged pressure overload precipitated the activation and transdifferentiation of cardiac fibroblasts, initiating a cascade of events leading to hypertrophic and fibrotic remodeling. This remodeling process culminated in myocardial stiffness, impaired early cardiac systolic dysfunction, and the eventual onset of systolic HF (1, 35). Early detection of these changes provides critical insights into disease progression and presents an opportunity to implement timely interventions aimed at mitigating or halting the pathological mechanisms driving cardiac dysfunction.

### 4.3 Relationship between EF1 and MF

An interesting finding of our study is the early functional decline, as measured by EF1, and the early pathophysiological changes, as evidenced by myocardial fibrosis (MF) development, occurring prior to the impairment of conventional measures such as strain and overall EF at the 2nd week post-operation. Pearson correlation coefficients revealed a significant relationship between EF1 and MF, with the correlation of MF with EF1 being stronger than that of EF. Although the correlation between EF1 and MF was also higher than that of the remaining parameters, the differences were small and not statistically significant. Notably, EF1 exhibited the highest AUC for the prediction of MF although it was only statistically different compared to EF and not to the remaining parameters, probably because of the small sample size.

EF1, which represents the volume change from the onset of systole to the point of peak aortic flow velocity, corresponds to the time of maximum myocardial contraction (17, 32). This novel measure is rooted in the biophysics of myocyte contraction, with this contraction usually occurring in the early phase of systole, peaking at the time of peak aortic flow velocity (20, 36). Moreover, alterations in afterload and adverse cardiac remodeling contribute to the shift of pressure-stress towards mid to late systole, leading to excessive late systolic wall stress. This mechanism likely underlies the impaired early left ventricular systolic function induced by pressure overload (17, 37).

Additionally, ECM plays a critical role in transmitting contractile forces and influencing cardiac systolic function (2, 33). Increased CSA of cardiomyocytes may lead to diminished oxygen diffusion to the hypertrophied myocardium, while myocardial interstitial fibrosis can result in reduced ventricular compliance (33, 38, 39). These factors collectively contribute to early systolic cardiac dysfunction in the left ventricle as reflected by the EF1.

### 4.4 Limitations

This study is subject to several significant limitations. Firstly, there are some limitations inherent to the MTAC model itself. A key constraint of the MTAC model used in our study—it effectively induces pressure-overload cardiac hypertrophy and fibrosis but does not replicate the complex comorbidities often present in human patients. Therefore, we must be careful when extrapolating findings to the clinical setting. Future studies are planned to extend this work by evaluating EF1 in MTAC models combined with additional comorbidities such as hypertension or renal dysfunction to better mimic human disease complexity. Additionally, it is important to consider that elevated afterload in the MTAC model may directly alter aortic valve flow parameters, such as increasing transvalvular gradients or contributing to functional regurgitation, independently of intrinsic cardiac dysfunction. While our Doppler assessments reflect global hemodynamic changes, including systolic performance, we cannot fully exclude the influence of afterload alone on the observed aortic valve flow patterns.

Secondly, there is potential measurement variability of EF1 related to operator-dependence in echocardiography. EF1 was measured using continuous Doppler imaging at the aortic valve and the two-dimensional biplane method. Conventional clinical measurements may be subject to greater variability. However, ICCs and Bland-Altman analysis indicated that EF1 had good reproducibility in this study. Furthermore, the TAVPF/R-R interval did not differ significantly between MTAC and sham groups rats, suggested that changes in EF1 were unlikely to be confounded by variations in aortic flow timing (Supplementary Table S4). These results clarify the reliability of EF1 as an echocardiographic parameter. In addition, we did not perform confirmatory phenotyping through serial sectioning with adjunctive histochemical stains (e.g., picrosirius red or α-SMA immunohistochemistry), which is a methodological limitation of the current study. However, this limitation underscores important directions for future work aimed at mechanistic characterization of fibrotic patterning and heterogeneity across myocardial layers and regions.

## 5 Conclusions

In conclusion, our study unveiled, for the first time, the association between MF and EF1 in a pressure overload induced cardiac dysfunction rat model. These findings suggested that EF1 served as a promising indicator for identifying MF at the early stages of cardiac dysfunction. Given its sensitivity to early contractile dysfunction, EF1 may be particularly useful in detecting subtle myocardial impairment in conditions such as ischemia-induced cardiomyopathy (characterized by early fibrosis), cardiac amyloidosis (with increased myocardial stiffness), and early-phase heart failure with preserved ejection fraction (HFpEF), where conventional EF may still appear within the normal range.

## Data Availability

The original contributions presented in the study are included in the article/Supplementary Material, further inquiries can be directed to the corresponding authors.
