# Supplementary material for: First-phase ejection fraction is associated with myocardial fibrosis in the pressure overloaded heart
Source: Front Cardiovasc Med. 2025 Jul 23;12:1603082. doi: 10.3389/fcvm.2025.1603082 (PMC12325372; doi:10.3389/fcvm.2025.1603082)
Supplement: Supplementary file 1 [file Datasheet1.pdf]

Supplemental material

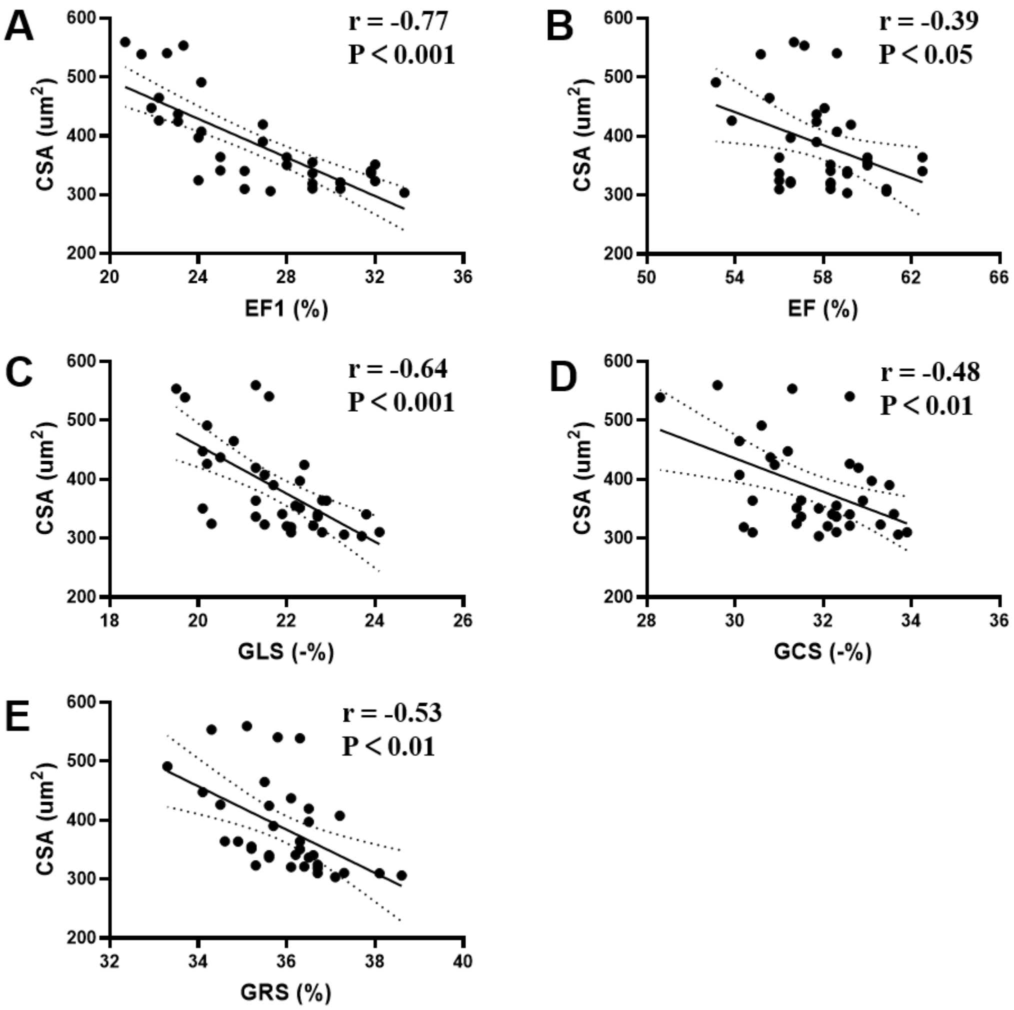

Supplementary Figure 1. Correlation analyses between CSA and EF1 (A), EF (B), GLS (C), GCS (D) and GRS (E) in MTAC group rats. CSA, cross-sectional area; EF1, first-phase ejection fraction; EF, left ventricular ejection fraction; GLS, global longitudinal strain; GCS, global circumferential strain; GRS, global radial strain. n = 35.

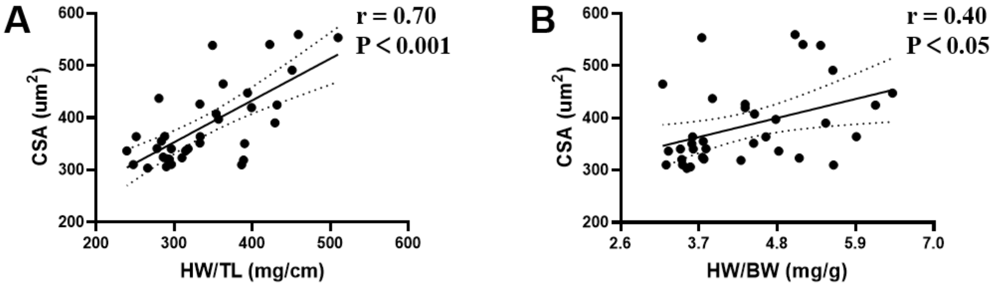

Supplementary Figure 2. Correlation analyses between CSA and HW/TL (A) and HW/BW (B) in MTAC group rats. CSA, cross-sectional area; HW/TL, heart weight to tibia length ratio; HW/BW, heart weight to body weight ratio. n = 35.

Supplementary Table 1. Multivariable linear regression analysis for MF associated with EF1 and CSA

Variable	β	95% CI	P value
EF1, %	-0.22	-7.78, 4.39	0.03
CSA, μm^2	0.76	-0.30, -0.01	<0.001

MF, myocardial fibrosis; EF1, first-phase ejection fraction; CSA, cross-sectional area; CI, confidence interval. n = 35.

Supplementary Table 2. Intra-observer and Inter-observer Variability

	ICC (95% CI)	Bias	Limits of agreement
MF, %			
Intra-observer	0.95 (0.83 – 0.99)	0.10	-1.42 – 1.62
Inter-observer	0.94 (0.79 – 0.98)	-0.21	-1.81 – 1.39
EF1, %			
Intra-observer	0.80 (0.40 – 0.95)	0.14	-4.26 – 4.55
Inter-observer	0.75 (0.31 – 0.93)	0.84	-5.34 – 7.01

EF1, first-phase ejection fraction; MF, myocardial fibrosis; CI, confidence interval; ICC, intraclass correlation coefficient. n = 10.

Supplementary Table 3. The statistical power of EF1 and MF

Parameter	Timepoint	Mean ± SD of MTAC rats (n = 7)	Mean ± SD of sham rats (n = 6)	Statistical power
EF1, %	Week 2	26.6 ± 1.3	30.0 ± 1.5	0.98
MF, %	Week 2	3.00 ± 0.72	1.62 ± 0.18	0.99

EF1, first-phase ejection fraction; MF, myocardial fibrosis; MTAC, minimally invasive transverse aortic constriction.

Supplementary Table 4. The ECG R-R interval and TAVPF of rats

Parameter	Time point	Mean ± SD of MTAC rats (n = 7)	Mean ± SD of sham rats (n = 6)	P value
R-R interval, ms	Week 0	174.4± 3.2	180 ± 11	0.84
	Week 1	180 ± 10	173.7 ± 3.3	0.76
	Week 2	177.6 ± 9.6	172.2 ± 3.8	0.88
	Week 3	179 ± 14	172.2 ± 6.2	0.59
	Week 4	185 ± 13	187.0 ± 6.8	> 0.99
TAVPF, ms	Week 0	42.6 ± 4.1	40.2 ± 4.4	0.86
	Week 1	44.2 ± 4.2	42.44 ± 6.7	0.95
	Week 2	42.1 ± 5.4	41.2 ± 3.5	> 0.99
	Week 3	43.9 ± 3.9	39.7 ± 2.8	0.36
	Week 4	44.5 ± 4.3	39.0± 2.2	0.13
TAVPF/R-R interval	Week 0	0.25 ± 0.03	0.23 ± 0.04	0.61
	Week 1	0.25 ± 0.03	0.24 ± 0.04	> 0.99
	Week 2	0.24 ± 0.02	0.24 ± 0.02	> 0.99
	Week 3	0.25 ± 0.03	0.23 ± 0.02	0.83
	Week 4	0.24 ± 0.03	0.21 ± 0.01	0.12

TAVPF, time to peak aortic valve flow; MTAC, minimally invasive transverse aortic constriction.
